# Overcoming unintended immunogenicity in immunocompetent mouse models of metastasis: the case of GFP

**DOI:** 10.1038/s41392-022-00929-9

**Published:** 2022-03-03

**Authors:** Christoph Schultheiß, Mascha Binder

**Affiliations:** Department of Internal Medicine IV, Oncology/Hematology, Martin-Luther-University Halle-Wittenberg, 06120 Halle, Saale Germany

**Keywords:** Cancer models, Genetic engineering

The applicability of immune-competent mice to model metastatic tumor progression is limited by the immunogenicity of exogenous proteins like GFP and firefly (ff) luciferase commonly used to track metastatic cells. A recent work published in *Cancer Cell* by Grzelak and colleagues now shows that development of mouse models that have been centrally tolerized to GFP can overcome this obstacle in transplantation models of metastasis.^1^

Despite major progress in cancer treatment, metastasis remains the key cause of treatment failure and cancer-related deaths worldwide. Metastasis formation is a hallmark of most malignant tumors that has been characterized as a multistep process of cancer cell dissemination from the primary tumor site followed by the seeding or formation of new tumor colonies in the same or distant organs. While some of the mechanistic principles underlying metastatic spread—like the epithelial–mesenchymal transition (EMT)—have been elucidated over the years, the process as a whole is still insufficiently understood and we currently lack a unifying conceptual framework that explains the multifaceted pathobiological variation of metastases relative to their primary tumor. For this reason, options for targeted therapy are scarce.^2^ This emphasizes the need to develop model systems which recapitulate the complex genomic and transcriptomic processes leading to initiation and progression of metastatic cell growth. The currently most widely used organism to model metastasis on the organismic level are xenografts or syngeneic mouse models using primary or cultured cells in an immunocompromised setting.^3^ Technically, the introduction and expression of reporter proteins like GFP is a prerequisite to monitor initiation and progression of metastases. Unfortunately, GFP is immunogenic and has been shown to influence growth and progression of metastasis.^4^ Since the immune system is involved in metastatic spread, this causes a serious technical problem limiting the validity of data derived from such models.

**Fig. 1:**
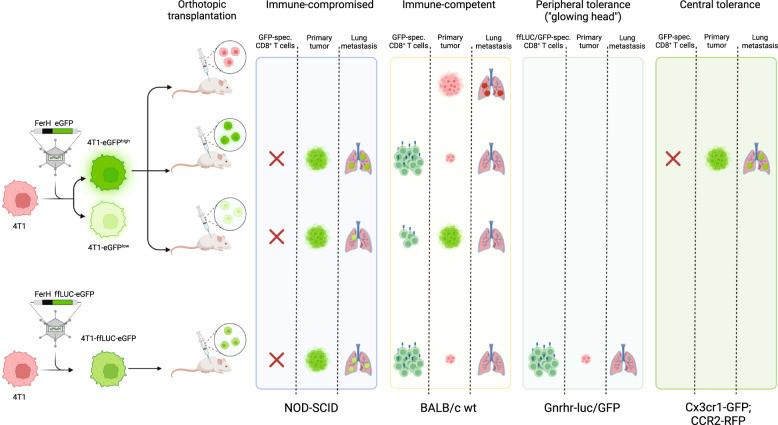
Unintended immunogenicity of GFP in immunocompetent mouse models of metastasis can be avoided by using models with central tolerance towards GFP. GFP-expressing metastatic 4T1 breast cancer cells form lung metastases after orthotopical transplantation in immunocompromised NOD-SCID mice. In immunocompetent BALB/c mice and a model of peripheral tolerance (Gnrhr-luc/GFP), formation of metastasis is prevented by a GFP-specific CD8^+^ T cell response independent of GFP expression level. In contrast, GFP-expressing 4T1 cells can form lung metastases while lacking a GFP-directed T cell response in a model of central tolerance towards GFP (Cx3cr1-GFP;CCR2-RFP). Created with BioRender.com

To address this issue, Grzelak and colleagues engineered a GFP-expressing variant of the murine 4T1 cell line, a highly metastatic triple-negative breast cancer cell line isolated from the mammary gland of BALB/c mice. When orthotopically implanting these cells into immunocompromised NOD-SCID mice, GFP^+^ metastases were readily detected in the lung, while this was not the case after implantation into immunocompetent syngeneic BALB/c mice (Fig. 1). Here, the lack of lung metastases correlated with tumor cell rejection, loss of GFP expression and a strong CD8^+^ T cell response against distinct GFP epitopes. However, dampening this T cell response by transplanting a 4T1 subline with decreased GFP expression allowed tumor growth at the injection site but was not sufficient to restore the metastatic potential of these cells to a level observed in the immunocompromised mice (Fig. 1).

Based on these observations and in line with reports that GFP expression in mice results in graft rejection and impacts formation and progression of metastais,^4^ the authors hypothesized that models which tolerize to the foreign antigen might be key to solve this problem. To test this hypothesis, the authors investigated whether extra-thymic expression or expression in professional antigen-presenting cells from birth, especially in the thymus, can tolerize mice to GFP. First, they used the “Glowing Head” (GH) mouse, which expresses a ffLuc-eGFP fusion protein in the anterior pituitary gland and testes, as model for peripheral tolerance and repeated the 4T1 transplantation assays including another 4T1 subline expression a ffLUC-eGFP fusion protein. However, GH mice also lacked imageable metastases in the lung, liver, brain and bone and mounted GFP-/ffLUC-specific CD8^+^ T cell responses as observed in wild-type mice (Fig. 1). In contrast, orthotopical transplantation of GFP-expressing 4T1 cells into *Cx3cr1-GFP;CCR2-RFP* mice that served as model for central tolerance by expressing GFP in monocytes, NK cells, brain microglia and dendritic cells did not elicit a GFP-specific CD8^+^ T cell response and allowed metastatic outgrowth as observed in immunocompromised mice and after transplantation of parental 4T1 cells (Fig. 1). Notably, the role of central tolerance for metastatic outgrowth of GFP-expressing cells was confirmed in another mouse strain expressing GFP mainly in the thymus (C57BL/6, *Aire-GFP*).

While this work elegantly presents a strategy for overcoming unintended immunogenicity in the 4T1 transplantation model, it also raises the question of the mechanistical underpinnings, since peripheral tolerance to ffLUC-eGFP could be induced in GH mice using a lung cancer transplantation model.^4^ It appears plausible that the source of the antigen and/or its processing and presentation are major determinants for effective induction of peripheral tolerance and not the action of distinct tolerance checkpoints that distinguish central (clonal deletion and clonal diversion) from peripheral (quiescence, ignorance, anergy, exhaustion) tolerance, at least for GFP epitopes. Of course, further work is warranted to test the generalizability of these findings in other GFP-based transgenics (especially in the context of MHC haplotypes) and to assess the relevance for further fluorophores.

A few other aspects of this study are noteworthy. One of them relates to the fact that GFP-directed CD8^+^ T cell responses preferentially blocked metastatic growth, while reduced GFP expression allowed local proliferation of injected tumor cells. This observation is interesting because it suggests an immunogenic threshold which allows these cells to escape the antitumor immune response at the primary site. This provides the compelling opportunity to use these scalable GFP-directed T cell responses as tool to study mechanisms of tumor cell dissemination and immune evasion of dormant tumor cells in the pre-metastatic niche. Moreover, this work may also have implications for other research questions including our interpretation and the future design of genome editing studies in murine and human systems since Cas9 has been shown to be immunogenic and targeted epitopes have been previously identified.^5^

Overall, Grzelak and colleagues show that central tolerization to GFP may help to overcome unintended immunogenicity in immunocompetent mouse models of metastasis. This work, therefore, delivers a relatively simple tool that can be of great importance for the worldwide community working to improve both our understanding of metastatic spread and develop strategies to therapeutically target this process.
